# End-to-End Autonomous Driving Decision Method Based on Improved TD3 Algorithm in Complex Scenarios

**DOI:** 10.3390/s24154962

**Published:** 2024-07-31

**Authors:** Tao Xu, Zhiwei Meng, Weike Lu, Zhongwen Tong

**Affiliations:** 1 National Key Laboratory of Automotive Chassis Integration and Bionics, Jilin University, Changchun 130015, China; xutao22@mails.jlu.edu.cn (T.X.); mengzw20@mails.jlu.edu.cn (Z.M.); tongzw22@mails.jlu.edu.cn (Z.T.); 2 School of Rail Transportation, Soochow University, Suzhou 215031, China

**Keywords:** autonomous driving, intelligent decision-making, complex scenarios, reinforcement learning, multiple critics

## Abstract

The ability to make informed decisions in complex scenarios is crucial for intelligent automotive systems. Traditional expert rules and other methods often fall short in complex contexts. Recently, reinforcement learning has garnered significant attention due to its superior decision-making capabilities. However, there exists the phenomenon of inaccurate target network estimation, which limits its decision-making ability in complex scenarios. This paper mainly focuses on the study of the underestimation phenomenon, and proposes an end-to-end autonomous driving decision-making method based on an improved TD3 algorithm. This method employs a forward camera to capture data. By introducing a new critic network to form a triple-critic structure and combining it with the target maximization operation, the underestimation problem in the TD3 algorithm is solved. Subsequently, the multi-timestep averaging method is used to address the policy instability caused by the new single critic. In addition, this paper uses Carla platform to construct multi-vehicle unprotected left turn and congested lane-center driving scenarios and verifies the algorithm. The results demonstrate that our method surpasses baseline DDPG and TD3 algorithms in aspects such as convergence speed, estimation accuracy, and policy stability.

## 1. Introduction

With the rapid advancement of automotive intelligence, autonomous driving has garnered significant attention from both the academic and industrial sectors [1,2]. Currently, the realization of autonomous driving generally involves two approaches: traditional sequential modular and end-to-end solutions [3,4]. Among them, traditional modular approaches tend to rely heavily on expert rules, while end-to-end imitation learning overly depends on the quality of the dataset, thereby limiting their effectiveness in addressing complex scenarios [5]. However, complex scenarios are often the gathering place for traffic accidents. For example, research from the U.S. Department of Transportation (Washington, DC, USA), based on an analysis of over 2 million accidents, indicates that the accident rate for left turns at intersections is nearly 20 times higher than that for right turns [6,7,8]. Meanwhile, vehicle roadway departure accidents are a significant safety concern, often resulting in severe injuries and fatalities [9,10]. Therefore, enhancing the decision-making capability of the ego vehicle is at the core of intelligent automotive technology [11,12,13]. End-to-end reinforcement learning, with its demonstrated excellent decision-making performance in complex scenarios, has become a hot topic in the field of autonomous driving [14].

In 2020, Wang et al. applied the DDQN algorithm to unprotected left turns [15]. However, the output is discrete actions, making it difficult to ensure safety and comfort during the driving process [6]. In 2022, Li et al. applied the DDPG algorithm with a continuous action space to left turn and straight through tasks at intersections [16]. In 2023, Ashwin et al. completed the task of lane keeping using the DDPG algorithm [17], which enables vehicles to accomplish tasks with higher safety and comfort through continuous actions. Yet, single-critic algorithms like DDPG suffer from overestimation, leading to inefficient task completion. Addressing these challenges, Fujimoto et al. constructed the TD3 algorithm by selecting the minimum value from a double-critic approach, and the algorithm’s overestimation problem is solved [18]. However, the TD3 algorithm itself encounters issues such as network underestimation, resulting in slow convergence and suboptimal policies [19,20].

To address the aforementioned issues, this paper proposes Triple Critics Average Maximization (TCAMD), an improved algorithm built upon the TD3 framework. Concurrently, we construct an end-to-end autonomous driving decision-making method based on the TCAMD algorithm. This method addresses the underestimation of the TD3 algorithm and improves the decision-making ability of autonomous vehicles in complex scenarios. By opting for a continuous action space as the output, this reinforcement learning (RL) algorithm demonstrates enhanced safety and comfort compared to algorithms with discrete action space. To validate its efficacy, the algorithm was rigorously evaluated in complex scenarios, including a multi-vehicle unprotected left turn scenario and a congested lane-center driving scenario. The contributions of this research are summarized as follows:

(1) To address the underestimation issue in the TD3 algorithm, this paper introduces a new single critic and incorporates TD3 weighting calculations to form a triple-critic structure. Additionally, inspired by TD3’s double-critic minimization approach, we propose a target maximization method that selects the maximum value between the old and new critics. These methods effectively address the underestimation problem.
(2) We adopt a multi-timestep averaging method to mitigate issues related to overestimation in the algorithm. Concurrently, we conduct a corresponding theoretical analysis of these methods to showcase their effectiveness.
(3) To enhance the decision-making capability of autonomous vehicles, we propose an end-to-end autonomous driving decision-making method based on the aforementioned improved algorithm. We conduct experiments in left turn and lane-center driving scenarios to verify its performance.

The remaining overall structure of this paper is as follows: Section 2 introduces fundamental concepts in reinforcement learning. Section 3 introduces in detail the significance of the two scenarios and their modeling. Section 4 introduces the improved algorithm. Section 5 details the experimental implementation and presents findings from experiments. Finally, Section 6 concludes with a summary and offers insights for future research directions.

## 2. Theoretical Background

In this section, we introduce the symbols and concepts pertinent to reinforcement learning algorithms. Moreover, we also explore the causes and hazards associated with overestimation and underestimation in target Q values.

### 2.1. Fundamental Concepts

**Figure 1:**
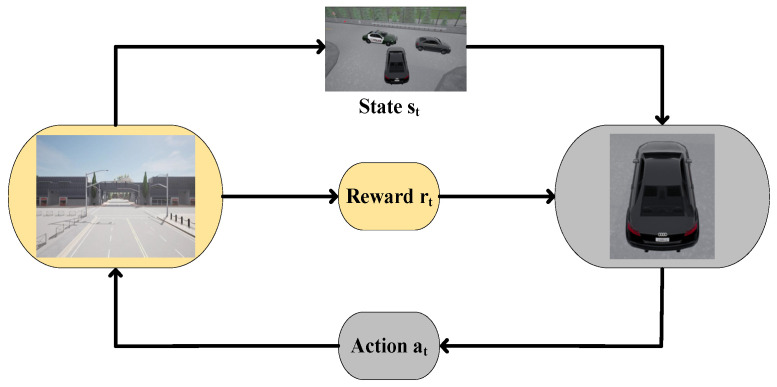
Interaction between ego vehicle and environment.

In Figure 1, a standard reinforcement learning process is shown, assuming the ego vehicle is in the current state *s_t_*. Subsequently, it makes a decision and executes a certain action *a_t_*. At this moment, the environment promptly provides reward feedback *r_t_*, while the state of the ego vehicle transitions to a new state *s_t_*_+1_. The process continues as a new action *a_t_*_+1_ is decided and executed in the state *s_t_*_+1_, forming a repetitive loop until an optimal policy that maximizes the expected cumulative discounted reward is obtained [21].

The cumulative discounted reward, also known as the return [22], is defined as follows:

(1) $$G_{t}=\sum_{k=0}^{\infty }\gamma^{k}R_{t+k}$$

In the formula, *G_t_* represents the cumulative discounted reward obtained at time *t*, and *γ* is the cumulative discount factor, ranging between 0 and 1. Subsequently, the action value function *Q*(*s,a*) can be derived from *G_t_* [23], defined as follows:

(2) $$Q(s,a)=E_{\pi }[G_{t}|(s_{t}=s,a_{t}=a)]$$

In the formula, *π* denotes the policy function, and *Q*(*s,a*) based on the policy *π* represents the expected return that the agent obtains [24]. Its recursive update rule is defined as follows [25]:

(3) $${Q(s}_{t}{,a}_{t}{)=R}_{t}+\gamma \underset{{(s}_{t+1}{,a}_{t+1}{)∼\rho }_{\pi }}{E}{[Q(s}_{t+1}{,a}_{t+1})]$$

In the specific calculation process, value function typically does not explicitly express the policy *π*. Consequently, in value iteration algorithms like DDPG and TD3, it is common to decouple the policy-evaluation and policy-improvement phases [26]. Subsequently, neural networks are employed to train the critic and actor networks of the algorithm [27]. The loss functions for these two networks are defined as follows:

(4) $$L^{C}(\theta )=\frac{1}{2}{\left(R_{t}+\gamma E[Q(s_{t+1},\pi (s_{t+1};\phi );\theta )]-Q(s_{t},a_{t};\theta )\right)}^{2}$$

(5) $$L^{A}(\phi )=-Q(s_{t},\pi (\cdot |s_{t};\phi );\theta )$$

In the formulas, *θ* and *ϕ*, respectively, represent the network parameters for the critic and actor networks. *L^C^*(*θ*) is the critic network’s loss function, typically computed as mean squared error and updated via gradient descent. *L^A^*(*ϕ*) represents the actor’s loss function, usually defined as the negative of the mean output of the critic network and updated through gradient ascent [28].

### 2.2. Overestimation and Underestimation of Target Q Values

#### 2.2.1. Target Q Value Overestimation

The target Q value overestimation was initially observed by Thrun et al. in the Q learning algorithm within discrete action space. The specific definition is as follows: When approximating the target Q value through function approximation, if the estimated value exceeds the true Q value, it is referred to as the overestimation phenomenon [29]. This phenomenon is often caused by the maximization of greedy policies, network noise, and imprecise estimation of target Q values [30]. The DDPG algorithm, which employs an actor–critic structure to generate continuous actions, does not explicitly involve a max operation. However, the single-critic structure of DDPG often leads to the overestimation phenomenon [17]. The overestimation of target Q values is prone to generating noise and high variance, resulting in increased estimation bias, decreased policy stability, and even divergence in policies that have already converged.

Regarding the overestimation issue prevalent in single-critic frameworks such as DQN and DDPG, in 2015 Van Hasselt et al. proposed the famous DDQN algorithm, which introduces independent networks for Q value estimation, to address the overestimation phenomenon of the traditional single network DQN [31]. In 2019, Fujimoto et al. proposed the famous TD3 algorithm. This algorithm introduces an additional critic network, which along with the original critic network takes the minimum value between them, effectively addressing the issue of overestimation in DDPG. Additionally, it smooths and regularizes the target policy while delaying the update of the policy network, thereby enhancing the overall performance of the algorithm. In recent years, the TD3 algorithm has emerged as one of the top-performing algorithms in the field of reinforcement learning [18].

#### 2.2.2. Target Q Value Underestimation

In recent years, the problem of underestimation in the TD3 algorithm has gradually garnered attention. The TD3 algorithm, as a state-of-the-art method in the field of reinforcement learning, addresses the overestimation phenomenon. However, the minimization operation itself introduces the risk of underestimation [30], which can lead to issues such as suboptimal policies and slow convergence. In 2023, Peng et al. proposed the SD3 algorithm, which employs the softmax operator and clips the action space to tackle the underestimation issue in the TD3 algorithm [32]. Furthermore, in 2024, Luo et al. adopted a method of averaging two critics, thereby alleviating the underestimation issue in the TD3 algorithm [33]. Therefore, to better understand the occurrence of underestimation and propose corresponding improvement policies, this section will demonstrate the phenomenon of underestimation in the TD3 algorithm. The following will involve derivation and explanation of the algorithm’s underestimation.

Assuming the target critic Q value estimate of the TD3 algorithm is denoted as *Q’_i_*, where *i =* {1,2}. The target estimate value *Q’_i_* and the true value *Q_true_* exhibit a certain error, defined as the error term *Y_i_ =* (*Q’_i_* − *Q_true_*), where *i =* {1,2}. These errors are independently and identically distributed within the range [−*μ*, *μ*], following a uniform distribution model. Since the true value *Q_true_* is unattainable, the TD3 algorithm employs the method of using double critics to estimate *Q_true_* [30], specifically expressed as

(6) $$Q^{\prime }(s_{t},a_{t})=R_{t}+\gamma \underset{i=1,2}{\min}{Q^{\prime }}_{i}(s_{t+1},\pi (s_{t+1}))$$

The next step involves substituting *Q_true_* into the equation above, resulting in the compound random variable *Z′* concerning the error term *Y_i_*:

(7) $$\begin{array}{l} Z^{\prime }=R_{t}+\gamma \underset{i=1,2}{\min}{Q^{\prime }}_{i}(s_{t+1},\pi (s_{t+1}))-(R_{t}+\gamma {Q^{\prime }}_{true}(s_{t+1},\pi (s_{t+1})))\\ =\gamma \underset{i=1,2}{\min}({Q^{\prime }}_{i}(s_{t+1},\pi (s_{t+1}))-{Q^{\prime }}_{true}(s_{t+1},\pi (s_{t+1})))\\ =\gamma \underset{i=1,2}{\min}(Y_{1},Y_{2}) \end{array}$$

Since the positive value *γ* does not affect the sign of the expectation, the sign of the expected value of the variable *Z′* aligns with the expectation of the error in the TD3 algorithm. Subsequently, we model the probability density and probability distribution function of *Y_i_*, and then obtain the probability distribution and expectation of the compound variable *Z′*. According to the definition of the uniform distribution, the probability density function of *Y_i_* is:

(8) $$f(x)=\left\{\begin{array}{ll} \frac{1}{b-a}, & a\leq x\leq b,\\ 0, & other. \end{array}\right.$$

In the equation, *a =* −*μ*, *b = μ*, and *x* represents each *Y* in *Y_i_*. By the relationship between the probability density and probability distribution, the probability distribution is

(9) $$F(x)=\left\{\begin{array}{ll} 0, & x<a,\\ \frac{x-a}{b-a}, & a\leq x<b,\\ 1, & x\geq b. \end{array}\right.$$

In the equation, assuming *Z’* = *γZ*, and since the random variables *Y_i_* in *Z*’ and *Z* are independently and identically distributed, the probability distribution of the compound variable *Z* is

(10) $$\begin{array}{l} F(z)=P(Z\leq z)=P(\underset{i=1,2,\ldots ,N}{\min}Y^{i}\leq z)\\ =1-P(\underset{i=1,2,\ldots ,N}{\min}Y^{i}>z)\\ =1-P(Y_{1}>z,\;\ldots \;,Y_{N}>z)\\ =1-P(Y_{1}>z)P(Y_{2}>z)\;\ldots \;P(Y_{N}>z)\\ =\left\{\begin{array}{ll} 0, & z\leq a,\\ 1-{\left(\frac{b-z}{b-a}\right)}^{N}, & a<z<b,\\ 1, & z\geq b. \end{array}\right. \end{array}$$

In the equation, for computational simplicity, the interval [−*μ*, *μ*] of the uniform distribution function is substituted into the formula to compute the expectation of the compound variable *Z*:

(11) $$\begin{array}{l} E(Z)=E(\underset{i=1,2,\ldots ,N}{\min}Y^{i})=\int_{-\infty }^{+\infty }[z\frac{dF(z)}{dz}]dz\\ =\int_{-\infty }^{u}0dz+\int_{-u}^{u}z(\frac{N}{b-a}){\left(\frac{b-z}{b-a}\right)}^{N-1}dz+\int_{u}^{+\infty }0dz\\ =\frac{N}{{\left(b-a\right)}^{N}}{\int_{-u}^{u}z(b-z)}^{N-1}dz\\ =-\frac{N-1}{N+1}u \end{array}$$

The TD3 algorithm employs double critics, resulting in two terms for *Y_i_*; hence, *N* equals 2. Substituting into Equation (11), we obtain E(*Z*) = (−1/3)*μ*. Since *Z′* and *Z* differ only by the positive factor *γ*, the expectation of Z′ is E(*Z′*) = (−1/3)*γμ*. This illustrates that the minimization operation in the TD3 algorithm can lead to an expected error being negative during updates, implying that the estimated target Q value is smaller than *Q_true_*, thus resulting in underestimation. This can lead to an excessively conservative exploration performance of the algorithm, slower convergence speed, and a tendency to converge to suboptimal policies.

## 3. Scenario Introduction and Modeling

In current reinforcement learning research, left turn scenarios are frequently oversimplified [16], and lane-center driving scenarios rely heavily on lane lines [17]. These simplified scenarios make it difficult to adequately evaluate the decision-making ability of the algorithm. Therefore, this paper constructs two complex scenarios on the Carla platform: a multi-vehicle unprotected left turn and lane-center driving in congested traffic. The following sections describe these scenarios and their modeling as reinforcement learning problems using Markov processes, and they are subsequently addressed with deep reinforcement learning algorithms.

### 3.1. Scenario Description

#### 3.1.1. Multi-Vehicle Unprotected Left Turn

**Figure 2:**
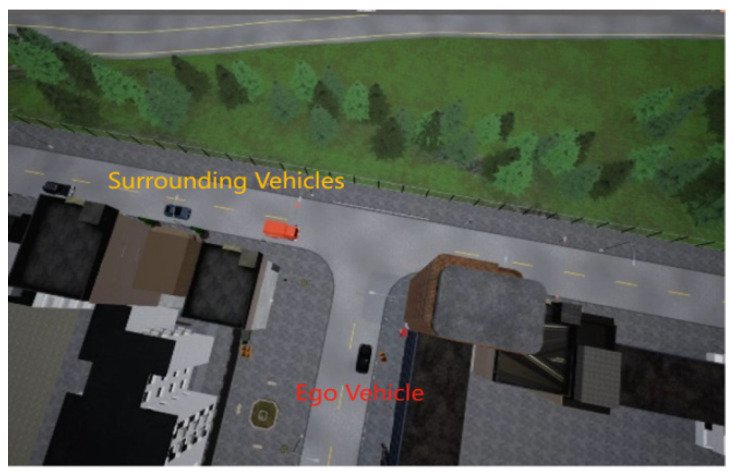
Multi-vehicle unprotected left turn scenario.

**Figure 3:**
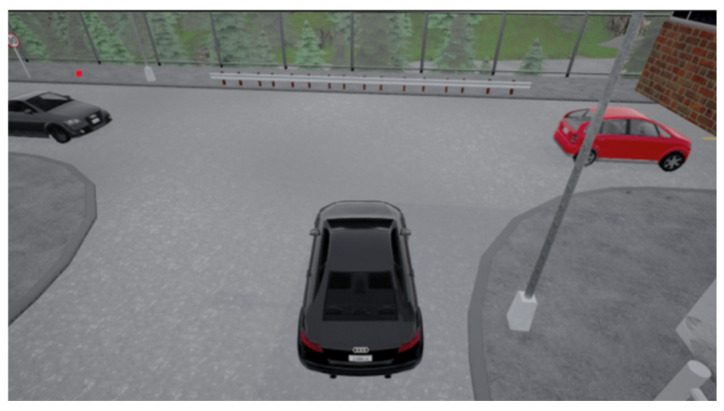
Vehicle flow with larger spacing.

**Figure 4:**
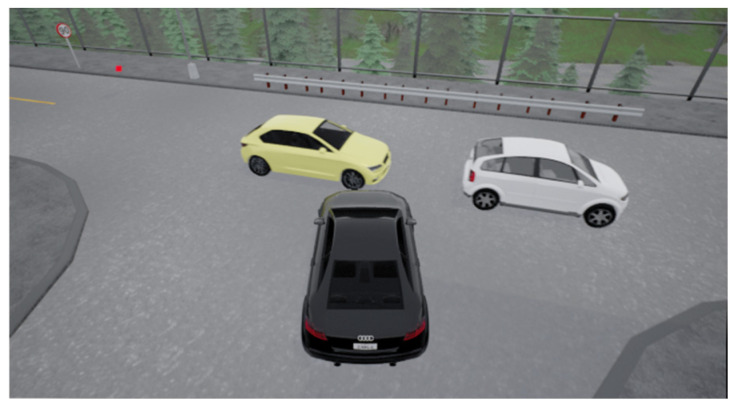
Vehicle flow with smaller spacing.

At the unsignalized intersection shown in Figure 2, the ego vehicle is tasked with making an unprotected left turn to reach the red target point. Meanwhile, several surrounding vehicles of random types will be laterally moving. These types include large trucks, medium-sized cars, and small tour buses, categorized based on volume and size. Consequently, the ego vehicle will pass smoothly when encountering a small tour bus but may cause a collision when confronted with a large truck. Meanwhile, the speed will be randomly initialized in the range of [4, 6] m/s, and it will vary while driving, leading to random variations in the distance between vehicles. As illustrated in Figure 3, when the ego vehicle faces a relatively large gap between vehicles, it needs to decide whether to proceed. In Figure 4, the ego vehicle encounters a smaller gap between vehicles, necessitating a decision on whether to wait. These sensible policy decisions heavily rely on accurate target Q value estimates. Additionally, surrounding vehicles do not yield to the ego vehicle, posing a greater challenge to the algorithm’s performance.

In this scenario, longitudinal planning takes precedence over lateral planning because executing longitudinal actions to avoid collisions is more effective. Therefore, the global path generated using Carla fulfills the requirements for lateral actions. This approach swiftly meets lateral scenario demands, freeing up attention and resources for crucial longitudinal control.

#### 3.1.2. Lane-Center Driving with Congestion Traffic

**Figure 5:**
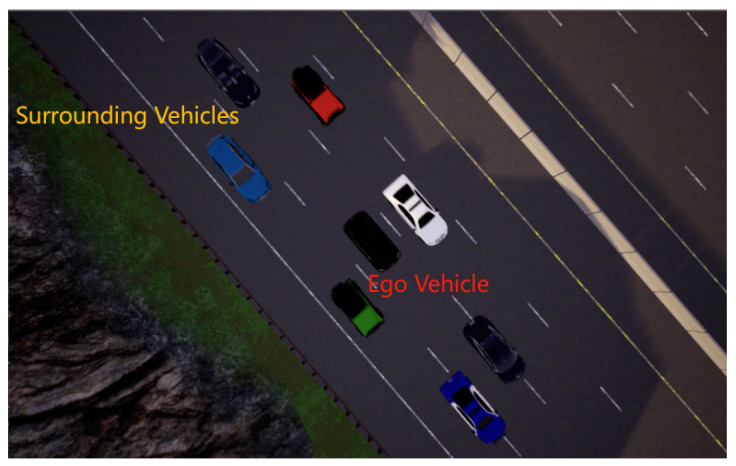
Lane-center driving with congestion traffic scenario.

As shown in Figure 5, the ego vehicle is surrounded by a group of vehicles whose speeds are randomly initialized within the range of [4, 6] m/s. The ego vehicle is unable to escape and can only follow the traffic flow by driving in the lane center. Surrounding vehicles will obscure lanes and other traffic signs, a common occurrence in the real world. Due to the close proximity between the ego vehicle and surrounding vehicles, there is a strict need for policy stability and accurate network estimation. Otherwise, there is a significant risk of collisions with the surrounding vehicles.

In this scenario, the primary objective is collision-free lane-center driving. Since smaller lateral distances often lead to collisions, lateral planning takes precedence over longitudinal planning. Therefore, this study employs the Intelligent Driver Model (IDM) to meet longitudinal motion requirements, thereby enabling more attention and concentration on optimizing lateral actions.

### 3.2. Scenario Modeling

This section will describe the state space, action space, and reward function.

#### 3.2.1. State Space S_t_

The state space *S_t_* for both scenarios consists of a set of state variables *s_t_*. A single monocular RGB camera is utilized to captures state information. Considering the limited information obtained from a single frame, the state information for the ego vehicle is extracted using the current and previous frames. Meanwhile, the captured images from the camera are resized to 80 × 60 to reduce the computational burden. The state variable *s_t_* is defined as:

(12) $$s_{t}=\{(p_{t-1})/255,(p_{t})/255\}$$

**Figure 6:**
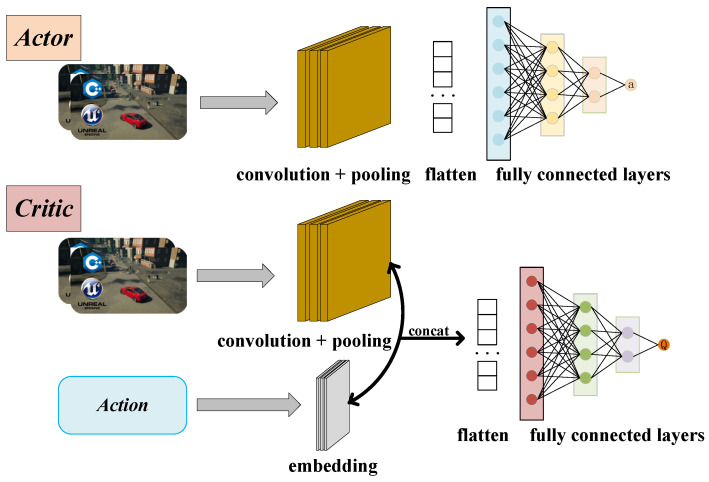
The inputs and outputs of actor and critic networks.

In the equation, *p_t_*_−1_ and *p_t_* represent the preprocessed raw camera data at the previous and current timesteps. As illustrated in Figure 6, the raw images are processed through convolution and pooling, flattened into a one-dimensional vector, and then fed into the actor–critic network. Additionally, the critic network will incorporate additional action vectors as input.

#### 3.2.2. Action Space A_t_

The action space *A_t_* consists of a set of action variables *a_t_*. These actions can include longitudinal actions such as throttle and brake, or lateral actions that control steering angle. Alternatively, they can be a combination of both longitudinal and lateral actions. In the left turn scenario, the algorithm selects throttle and brake as the outputs, and the expression for *a_tl_* is:

(13) $$-1\leq a_{tl}\leq 1$$

The above expression indicates that the magnitude of *a_tl_* can continuously change between −1 and 1. Values less than 0 represent deceleration, while values greater than or equal to 0 represent acceleration.

In the lane-center driving scenario, the algorithm selects the steering wheel angle as the output, and the expression for *a_tl_* is:

(14) $$-3\lambda \leq a_{tc}\leq 3\lambda$$

The above expression indicates that the magnitude of the ego vehicle’s output action *a_tc_* can continuously vary between −3*λ* and 3*λ*, where *λ* is a proportional adjustment factor with a value of 1. When *a_tc_* is less than 0, it indicates a left turn, and when greater than 0, it indicates a right turn.

Specifically, the action space in this paper is continuous, offering a distinct advantage over algorithms employing discrete action space. Discrete actions typically discretize longitudinal and lateral actions into arrays such as [−1, −0.5, 0, 0.5, 1] and [−3, −1, 0, 1, 3], respectively [6,34]. These discrete arrays can lead to undesirable vehicle behaviors such as sudden acceleration, abrupt braking, and significant lateral swings. Moreover, managing a large number of predefined actions significantly increases computational load. Therefore, the continuous action algorithm adopted in this study holds greater practical value compared to traditional discrete action algorithms.

#### 3.2.3. Reward Function

The reward functions for the two scenarios are defined as follows:

(15) $$R_{left}=a\times R_{goal}+b\times R_{count}+c\times R_{col}+d\times R_{speed}+e\times R_{ttc}$$

(16) $$R_{cong}=\alpha \times R_{goal}+\beta \times R_{col}+\tau \times R_{steer}$$

In the left turn scenario, the *R_left_* has the weights *a*, *b*, *c*, and *d* with specific values of 10, 100, −100, 0.5, and 0.1, respectively. *R_goal_*, *R_count_*, and *R_col_* are Boolean variables. *R_goal_* represents the reward for successfully completing the task, *R_count_* signifies the penalty for exceeding time limits, and *R_col_* denotes the penalty for collisions. Notably, *R_goal_* and *R_count_* impact the algorithm’s convergence and traffic efficiency, while *R_col_* influences the algorithm’s safety. The logic for the values of these three variables is similar; if the corresponding event occurs, the value is set to 1; otherwise, it is 0. *R_speed_* represents the speed reward concerning the desired velocity, which influences traffic efficiency. Simultaneously, the risk–reward term *R_ttc_* is designed by incorporating the Time to Collision (TTC) index. The formula for the TTC index is defined as follows:

(17) $$TTC=\frac{d_{\mathrm{rel}}}{v_{\mathrm{rel}}}$$

where *d_rel_* is the relative distance between the ego vehicle and the closest surrounding vehicle at the current moment, and *v_rel_* is the relative velocity between the ego vehicle and the closest surrounding vehicle.

In the lane-center driving scenario, the reward function *R_cong_* is defined as follows, where *α*, *β*, and *τ* are the weights for each term and are set to 10, −10, and −3. *R_goal_* and *R_col_* are Boolean variables, and the logic for assigning values is similar to that in the left turn scenario. *R_steer_* is the steering reward, defined as the absolute value of the change in steering wheel angle and aiming to guide the vehicle and enhance comfort. Existing RL lane-keeping research often heavily relies on lane lines in the reward function, enforcing adherence to the centerline rather than learning true centering policies. However, in this study, the ego vehicle operates in an environment surrounded by other vehicles, making it impossible to obtain information such as lane lines and traffic signs. Consequently, the reward function excludes such information, prompting the ego vehicle to interact dynamically with surrounding vehicles, developing effective lane-keeping policies. Complex scenarios and sparse rewards present challenges for the algorithm’s target estimation, policy stability, and overall performance metrics.

## 4. Algorithm Introduction

**Figure 7:**
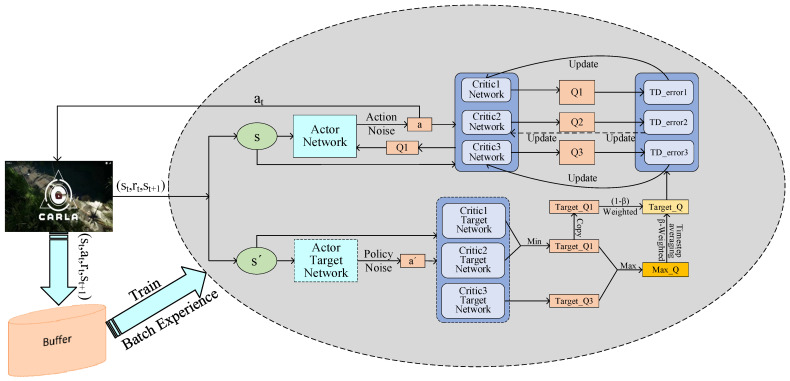
TCAMD algorithm framework.

As shown in Figure 7, the TCAMD algorithm forms a triple-critic structure by introducing a new single critic into the TD3 algorithm. Additionally, the algorithm further addresses the underestimation problem by maximizing the target, and adopts a multi-timestep method to deal with the policy instability problem brought by new critics. The following sections will provide a detailed introduction.

### 4.1. TCD Algorithm with Triple Critics

To address the issue of Q value underestimation in the TD3 algorithm, a new critic network is introduced, transforming the double critic TD3 algorithm into the Triple Critics Deep Deterministic Policy Gradient (TCD) algorithm. The update formula is as follows:

(18) $$y=r+\gamma [(1-\beta )\underset{i=1,2}{\min}{Q^{\prime }}_{i}(s^{\prime },\pi (s^{\prime }))+\beta {Q^{\prime }}_{3}(s^{\prime },\pi (s^{\prime }))]$$

where *β* is the weighting factor for the new critic, ranging from 0 to 1. Setting *β* to 0 corresponds to the TD3 algorithm, while setting *β* to 1 corresponds to the DDPG algorithm. Specifically, the original double-critic-estimated expectation is E_TD3_ = (−1/3)*γμ*. By replacing the *β*-weighted part with the new critic, assuming the new critic’s estimated expectation is *α*, the estimated expectation of the TCD algorithm becomes:

(19) $$E_{DDPG}=\alpha >E_{TCD}=(1-\beta )(-1/3\gamma u)+\beta \alpha >E_{TD3}=-1/3\gamma u$$

The expected value *α* in single-critic algorithms like DDPG is usually higher compared to the double critic of TD3 [23]. Introducing a new single critic can therefore address the issue of underestimation in the TD3 algorithm. However, it also makes the algorithm susceptible to overestimation. To mitigate this, we assign a relatively small *β* weighting to the new critic.

### 4.2. TCMD Algorithm with Maximization of the Target

Through literature review and experiments, it was observed that single critics are more prone to overestimation compared to TD3, but not invariably. This implies that the problem of underestimation in the TD3 algorithm has not been completely resolved, and there is still room for improvement. Building on this observation, this study draws inspiration from the TD3 algorithm’s approach of selecting the minimum value among double critics. Based on TCD, we adopt a method for selecting the maximum value between the double critics and the new critic, thereby replacing the newly introduced single critic. This results in the TCMD (Triple Critics Maximization Deep Deterministic Policy Gradient) algorithm, with the following specific formula:

(20) $${Q^{\prime }}_{3}(s^{\prime },\pi (s^{\prime }))=\max\{{Q^{\prime }}_{3}(s^{\prime },\pi (s^{\prime })),\underset{i=1,2}{\min}{Q^{\prime }}_{i}(s^{\prime },\pi (s^{\prime }))\}$$

The formula takes the maximum value between the output of the new critic *Q’*_3_ and the double critics, thereby further addressing the underestimation issue in the TD3 algorithm.

### 4.3. TCAMD Algorithm with Multi-Timestep Averaging

However, weighting and maximizing multiple critics can potentially lead to overestimation of Q value. This can result in unstable policies and reduced accuracy in Q value estimates. To mitigate these issues, this paper adopts the method of multi-timestep averaging, proposing the TCAMD algorithm (Triple Critics Average Maximization Deep Deterministic Policy Gradient).

The algorithm averages the outputs of the new critic *Q’*_3_ from the previous K − 1 timesteps and the current timestep. This method aims to further address the overestimation problem in the algorithm. The update formula is as follows:

(21) $${Q^{\prime }}_{3}(s^{\prime },\pi (s^{\prime }))=\frac{1}{K}\sum_{i=1}^{K}{Q^{\prime }}_{3}(s^{\prime },\pi (s^{\prime });\theta_{t+1-i}^{\prime })$$

where *θ’* represents the network parameters of the target critic network, and parameter K indicates the number of timesteps considered for averaging, set to 5 in this paper. Adopting multi-timestep averaging smooths the variance and fluctuations in the algorithm’s Q values and policies, thereby enhancing stability.

## 5. Results and Discussion

This section provides detailed explanations of the algorithm implementation, conducts ablation experiments, and discusses the results of scenarios involving left turn and lane-center driving.

### 5.1. Experimental Setup

The algorithm parameters are detailed in Table 1. Throughout this study, we maintained consistency in the experimental parameters to ensure a fair comparison of algorithm performance.

### 5.2. Ablation Experiments

This section compares the improved algorithm with DDPG and TD3 through comparative experiments. Additionally, we explore the effectiveness of each step in the algorithm improvements proposed in this study. Throughout the experiments, we evaluate the algorithm’s performances using metrics such as the convergence speed, average reward in the mid- and late terms, policy stability, success rate, time consumption, and driving rounds. We present data and metrics visually through plots, smooth the reward graph using a Gaussian approach, and organize detailed metric data in tables.

#### 5.2.1. Reward Curve and Converging Episodes

**Figure 8:**
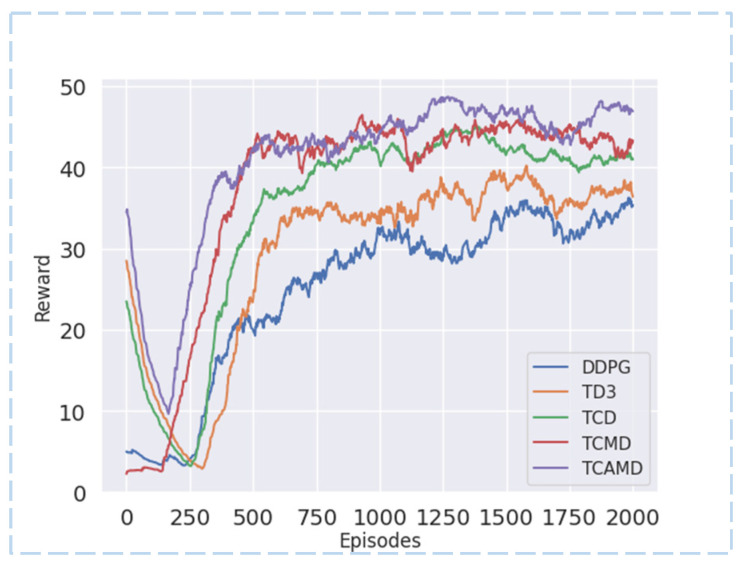
Reward curve for left turn scenario.

**Figure 9:**
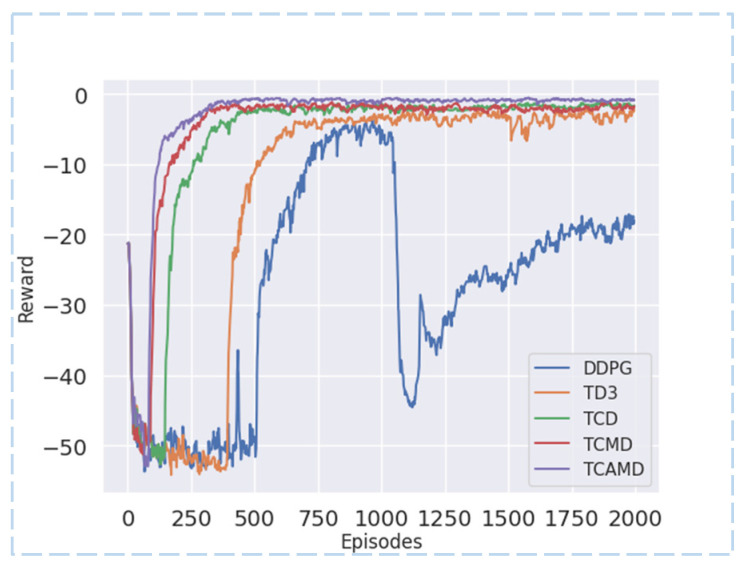
Reward curve for lane-center driving scenario.

From Figure 8 and Figure 9 and Table 2, the TCAMD algorithm demonstrates the fastest convergence speed and achieves the highest reward acquisition in both scenarios. The convergence speed index is determined by the episodes from the start to convergence. Faster convergence indicates stronger learning ability. Regarding reward values, early exploration in RL introduces stochasticity that can heavily influence the global average rewards in the initial phases. Therefore, this study focuses on mid-term (data after the first thousand episodes) and late-term (last five hundred episodes) average rewards to assess the algorithm’s reward acquisition capability. Higher reward acquisition indicates higher estimation accuracy of the algorithm and a better-fitted policy.

In the left turn scenario, both the DDPG and TD3 algorithms exhibit slower convergence speeds and lower reward values. The introduction of a new critic in the TCD algorithm significantly improves convergence speed and reward acquisition. Subsequently, the TCMD algorithm incorporates target maximization enhancements, further boosting the convergence speed and reward acquisition capabilities. Unfortunately, it exhibits variance fluctuations and a decline in policy stability. The TCAMD algorithm adopts a multi-timestep averaging method, resulting in the fastest convergence speed and highest reward values among all algorithms while also improving policy stability. In the left turn scenario, TCAMD demonstrates substantial improvements over the DDPG algorithm, achieving an approximately 73.54% increase in convergence speed and respective increases of 42.27% and 35.78% in mid- and late-term rewards. Compared to the TD3 algorithm, TCAMD demonstrates an approximately 57.05% increase in convergence speed, along with respective increases of approximately 26.40% and 25.52% in mid- and late-term reward acquisition. Overall, the TCAMD algorithm achieves significant enhancements across various performance metrics.

In the lane-center driving scenario, both the DDPG and TD3 algorithms continue to exhibit poor performance. Even the DDPG algorithm struggles to converge in the late term. This is attributed to its single-critic structure, which is prone to overestimation, leading to a divergence in policy. Additionally, the lane-center driving scenario is more susceptible to collisions. The TD3 algorithm demonstrates suboptimal policy performance in this context. While the TCD algorithm surpasses baseline algorithms, there remains room for improvement. The TCMD algorithm improves convergence speed compared to TCD but experiences policy instability due to higher variance. In contrast, the TCAMD algorithm achieves optimal performance by leveraging the strengths of both TCD and TCMD. Compared to the DDPG algorithm, TCAMD shows an approximately 63.21% improvement in convergence speed and successfully accomplishes the centering task where DDPG fails. Compared to TD3, TCAMD demonstrates approximately 57.54%, 73.50%, and 74.21% enhancements in convergence speed, mid-term reward acquisition, and late-term reward acquisition, respectively. These results showcase the outstanding task completion capabilities of the TCAMD algorithm.

#### 5.2.2. Mean and Standard Deviation of Rewards

**Figure 10:**
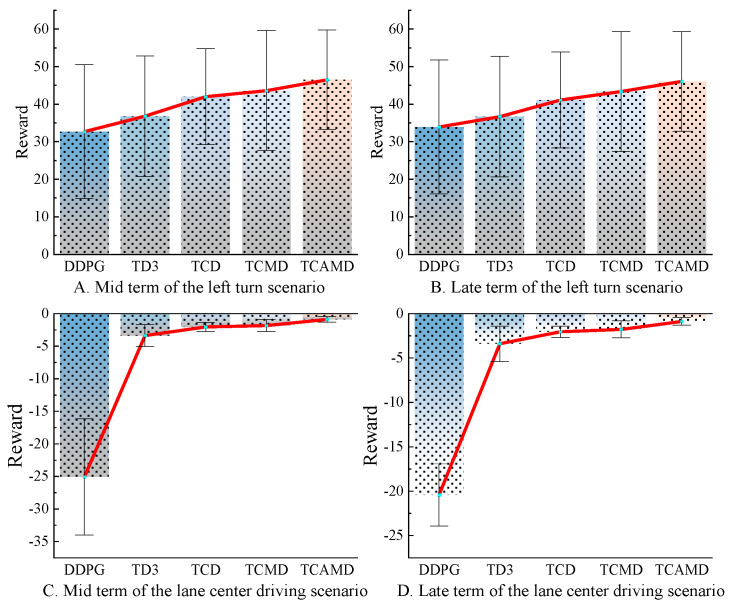
Bar chart of mid- and late-term metrics in the algorithm.

In Table 3 and Table 4, and Figure 10, AR denotes the average reward, and SD signifies standard deviation. Figure 10A,B show interval histograms of mid- and late-term rewards for the left turn scenario, while Figure 10C,D show interval histograms of mid- and late-term rewards for the lane-center driving scenario. Mean values are shown in blue, connected by red lines, with error bars indicating standard deviation to reflect the algorithmic policy stability. Smaller standard deviation values indicate improved policy stability. Moreover, each graph exhibits an upward trend, illustrating the effective enhancement of algorithm performance with each iterative improvement step.

As shown in Figure 10A,B, the TCAMD algorithm demonstrates higher average rewards in the left turn scenario compared to all other algorithms, accompanied by a relatively low standard deviation. This indicates that TCAMD exhibits high estimation accuracy, resulting in a superior-quality and stable fitted policy. Specifically, in the mid- and late terms of the left turn scenario, the TCAMD algorithm reduces the standard deviation by 25.47% and 18.00%, respectively, compared to DDPG. Compared to TD3, these reductions are 17.39% and 3.57%, respectively.

In the lane-center driving scenario, as shown in Figure 10C,D, TCAMD shows a 19.5% reduction in the standard deviation compared to DDPG in the mid-term. However, due to policy divergence in the late term, DDPG exhibits an excessively large standard deviation, making direct comparison challenging. Meanwhile, compared to TD3, TCAMD demonstrates reductions of 15.05% and 16.82% in the standard deviation for the mid- and late terms, respectively. These results highlight that the improved algorithm has strong stability and generalization in challenging scenarios.

It is noteworthy that the TCMD algorithm exhibits a superior reward acquisition capability than both the TCD and baseline algorithms. It occasionally approaches the policy performance achieved by the TCAMD algorithm. However, its policy stability appears to be weaker, as evidenced by the higher standard deviation indicated by the error bars. Therefore, multi-timestep averaging is introduced in the TCAMD algorithm to enhance policy stability. Moreover, the standard deviation of TCAMD increases in the late term compared to its own mid-term standard deviation. However, this increase is benign and stems from the algorithm’s continuous improvement in reward acquisition ability, as shown in Figure 1. This is different from the increase in standard deviation seen in the TCMD algorithm, which results from fluctuations in reward decline. Throughout, the standard deviation values of TCAMD consistently outperform those of the two baseline algorithms.

#### 5.2.3. Heatmap of Success Rates

**Figure 11:**
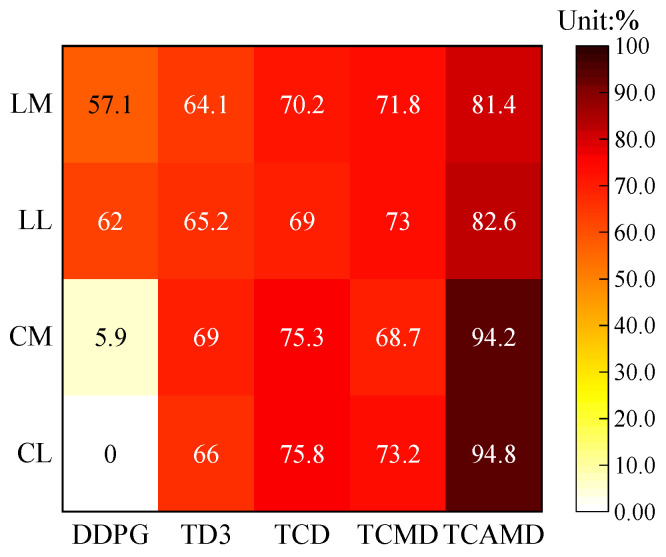
Heatmaps of success rates in the mid- and late terms for the two scenarios.

Figure 11 presents heatmaps illustrating the success rates of five algorithms during the mid- and late terms in both scenarios. LM and LL denote the mid- and late terms in the left turn scenario, while “CM” and “CL” denote the mid- and late terms in the lane-center driving scenario. The success rate is a crucial safety metric for algorithms, defined as the number of completed tasks divided by the total number of runs.

The TCAMD algorithm consistently achieves the highest success rate across all scenarios and time periods, showcasing its exceptional task completion capability. In contrast, the DDPG algorithm shows relatively lower success rates, particularly in the lane-center driving scenario where it exhibits significant declines and occasional task failures due to high variance and policy instability. Meanwhile, the TD3 algorithm performs moderately in both scenarios, serving as a reliable baseline. However, its susceptibility to underestimation limits its potential for achieving a significant improvement in success rates. The TCD algorithm shows marked improvement compared to baseline algorithms, indicating that the introduction of a new critic effectively addresses the underestimation issue inherent in the TD3 algorithm. However, in the lane-center driving scenario, the TCMD algorithm experiences reduced success rates due to amplified policy instability. By combining the strengths of various approaches, TCAMD emerges as the optimal solution, achieving the highest success rates overall.

#### 5.2.4. Refinement Metrics of Left Turn and Lane-Center Driving Scenarios

**Figure 12:**
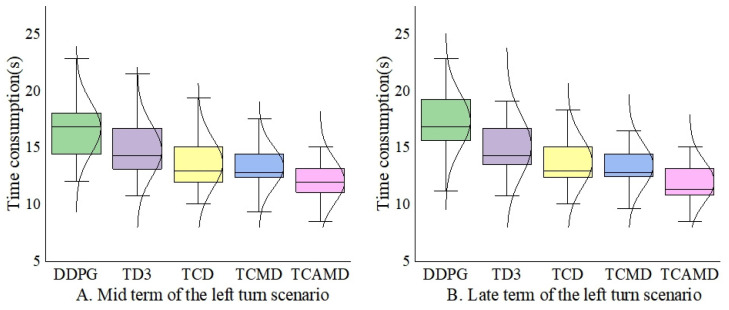
Box plot and distribution of time consumption in the left turn scenario.

**Figure 13:**
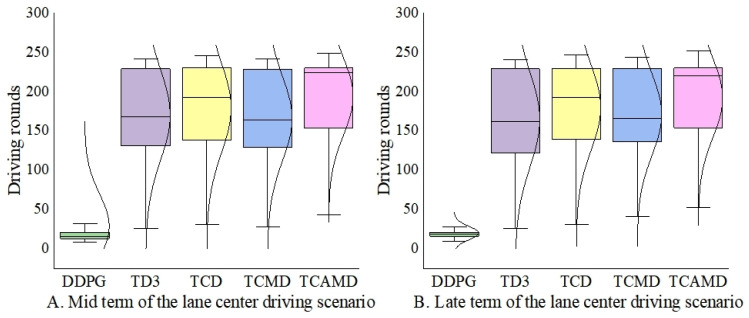
Box plot and distribution of driving rounds in the lane-center driving scenario.

As shown in Figure 12 and Figure 13, this paper introduces specific metrics tailored to each scenario’s characteristics. In Figure 12A,B, the mid- and late terms’ time consumption for successfully completed task rounds in the left turn scenario is presented. A smaller value for this metric indirectly signifies higher efficiency in algorithmic vehicle traversal. In Figure 13A,B, respectively, illustrate continuous driving rounds for the algorithm during the mid- and late terms in the lane-center driving scenario. Since the ego vehicle is surrounded in the centered scenario, the focus shifts from the travel time efficiency to the sustained driving capability. The driving rounds will reflect the ability to drive in the center. Therefore, longer continuous driving rounds indirectly indicate stronger safety and task completion capabilities of the algorithm.

Figure 13A,B illustrate that the TCAMD algorithm proposed in this paper sustains the longest duration, highlighting its exceptional estimation accuracy and policy stability. In contrast, both the DDPG and TD3 algorithms exhibit inferior performance, with their numerical distributions being noticeably skewed towards lower values. The TCMD algorithm tends to overestimate more than the TCD algorithm. In response, the TCAMD algorithm incorporates multi-timestep averaging to effectively enhance policy stability.

## 6. Conclusions

To address the issue of underestimation in the TD3 algorithm and the challenges of the ego vehicle’s limited decision-making ability, this paper proposes the TCAMD algorithm with a triple-critic network structure. By introducing new critic weights and target maximization methods, we effectively resolve the underestimation problem in TD3. Moreover, we enhance stability by controlling the weight β and adopting multi-time-step averaging. Subsequently, expanding on the TCAMD algorithm, we propose an end-to-end autonomous driving decision-making method to enhance ego vehicle decision-making capabilities. Theoretical derivations and experimental results confirm that the TCAMD algorithm surpasses DDPG and TD3 in terms of convergence speed, reward acquisition, security, and traversal efficiency.

Following that, in response to problems in existing studies with oversimplification in the left turn scenario and the excessive dependence on lane lines in the lane-center driving scenario, we construct more complex scenarios using the Carla platform for conducting experiments. The aim is to enhance the challenge for algorithms and increase the practical significance of the research.

This paper proposes the TCAMD algorithm, which falls within the field of online reinforcement learning. Due to the security concerns that come with online interaction, it has not yet been implemented in engineering applications. However, recent breakthroughs in offline reinforcement learning have provided possibilities for its practical implementation. Anticipating future developments, we will try to validate and deploy this algorithm in real-world vehicles using offline reinforcement learning.

## Figures and Tables

**Table 1:** Table of parameter configurations.

Parameter	Value
Hidden	256
*γ*	0.95
Tau	1 × 10^−3^
Capacity	38,400
Batch size	64
Actor learning rate	2 × 10^−4^
Critic Learning rate	5 × 10^−4^
*β*	0.05
K	5
Experimental setup	Ubuntu 20.04, NVIDIA RTX 4060

**Table 2:** Converging episodes.

Algorithm	DDPG	TD3	TCD	TCMD	TCAMD
Left turn	979	603	519	318	259
Lane-center driving	780	676	399	336	287

**Table 3:** Indicators in the left turn scenario.

Algorithm	DDPG	TD3	TCD	TCMD	TCAMD
Mid-term AR	32.66	36.77	41.97	43.57	46.47
Late-term AR	33.91	36.68	41.08	43.35	46.05
Mid-term SD	17.83	16.08	12.77	16.49	13.28
Lid-term SD	16.87	14.34	12.74	16.11	13.83

**Table 4:** Indicators in the lane-center driving scenario.

Algorithm	DDPG	TD3	TCD	TCMD	TCAMD
Mid-term AR	−25.10	−3.37	−2.06	−1.85	−0.89
Late-term AR	−20.43	−3.41	−2.05	−1.79	−0.87
Mid-term SD	8.92	1.69	0.71	0.92	0.44
Lid-term SD	3.50	1.99	0.62	0.95	0.43

## Data Availability

The data has been included in the article.
